# A novel probabilistic approach to generating PTV with partial voxel contributions

**DOI:** 10.1088/1361-6560/aa6b90

**Published:** 2017-05-22

**Authors:** H S Tsang, C P Kamerling, P Ziegenhein, S Nill, U Oelfke

**Affiliations:** pmbaa6b901 Joint Department of Physics, The Institute of Cancer Research and The Royal Marsden NHS Foundation Trust, London SM2 5NG, United Kingdom; henry.tsang@icr.ac.uk

**Keywords:** radiotherapy, treatment planning, margins

## Abstract

Radiotherapy treatment planning for use with high-energy photon beams currently employs a binary approach in defining the planning target volume (PTV). We propose a margin concept that takes the beam directions into account, generating beam-dependent PTVs (bdPTVs) on a beam-by-beam basis. The resulting degree of overlaps between the bdPTVs are used within the optimisation process; the optimiser effectively considers the same voxel to be both target and organ at risk (OAR) with fractional contributions. We investigate the impact of this novel approach when applied to prostate radiotherapy treatments, and compare treatment plans generated using beam dependent margins to conventional margins. Five prostate patients were used in this planning study, and plans using beam dependent margins improved the sparing of high doses to target-surrounding OARs, though a trade-off in delivering additional low dose to the OARs can be observed. Plans using beam dependent margins are observed to have a slightly reduced target coverage. Nevertheless, all plans are able to satisfy 90% population coverage with the target receiving at least 95% of the prescribed dose to $D_{98\%}$.

## 1. Introduction

Geometric uncertainties present in external beam radiation therapy using photons arise from various sources, for example organ motion and set up uncertainties. These uncertainties result in deviations between the planned dose distribution and the dose distribution that is physically delivered to the patient. If this is not accounted for, underdosage of the clinical target volume (CTV) or overdosage of organs at risk (OARs) can occur.

Current clinical practice employs the use of safety margins to define a planning target volume (PTV), to ensure to a clinically acceptable probability that sufficient dose is delivered to the CTV. A larger PTV implies a higher probability to achieve sufficient dose coverage. However, the size of the PTV is limited by the increase in normal tissue toxicity due to a larger irradiation volume.

Research related to uncertainties in radiation therapy is motivated by the unavoidable conflict between required target coverage probability and the amount of radiation delivered to the patients’ healthy tissue. We can categorise this research into either reducing the magnitude of the uncertainties, or finding different means of accounting for these uncertainties within the treatment planning process. Regarding the reduction of the uncertainties’ magnitude, recent publications include the use of internal markers in online verification to reduce set up uncertainties (McNair *et al* 2008), tracking the target using multi-leaf collimators to reduce uncertainties due to organ motion (Fast *et al* 2014, Colvill *et al* 2015, Fast *et al* 2016), and advanced organ segmentation using MRI to CT deformable registration to reduce delineation uncertainties (Khoo and Joon 2006, Hanvey *et al* 2012). On the treatment planning front, various methods have been proposed that forgo the use of margins. Some of the proposed techniques include the optimisation of the expectation value of the treatment objectives (Unkelbach and Oelfke 2004), worst case optimisation (Pflugfelder *et al* 2008), scenario based planning (Fredriksson and Bokrantz 2015), and CTV coverage based optimisation (Gordon and Siebers 2009, Sobotta *et al* 2010, Xu *et al* 2014).

The most common technique used in current clinical practise to determine the required size of the CTV to PTV margin is the recipe proposed by Van Herk *et al* (2000), shown in (1). This margin recipe focusses on the dose coverage of the CTV, accounts only for the geometric shifts of the CTV, does not consider the presence of rotations and deformations of the CTV and ignores any impact on OARs. The uncertainties for geometric shifts are further separated into two types: preparation and execution uncertainties.

Treatment execution uncertainties vary from treatment to treatment, and can be modelled as blurring of the cumulative dose distribution. These are often referred to as random uncertainties. Treatment preparation uncertainties, on the other hand, affect all treatment fractions in the same way, but vary stochastically across the patient population. These are often referred to as systematic uncertainties, and can be modelled as displacements of the CTV relative to the cumulative (blurred) dose distribution. The respective margins take the form:

1 \begin{align*} {\rm Margin} = \alpha \Sigma + \beta \left(\sqrt{\sigma^2_r + \sigma^2_p} - \sigma_p \right) \label{eq:marginRecipe} \nonumber \end{align*}

Here, $\Sigma$ and $\sigma_r$ are the standard deviations of the systematic and random uncertainties, characterised by their respective Gaussian distributions; $\sigma_p$ refers to the size of the beam penumbra. The coefficients *α* and *β* are found by evaluating the relevant probability density functions, such that the bounding volumes defined by the coefficients result in the specified probability coverage. To achieve a patient population coverage of 90% where at least 95% of the prescribed dose is delivered to the CTV, the resulting coefficients are $\alpha=2.50$ and $\beta=1.64$.

This work aims to address one limitation of conventional margin recipes: the assumption that a perfectly conforming dose distribution can be delivered to the patient. In reality, photon beam radiation deposits exponentially less dose with depth, and whilst the lateral fall-off is much sharper, a gradient (or penumbra) is still present. In other words, small displacements in beam direction result in small deviations from the planned dose, whereas displacements perpendicular to beam direction can result in severe underdosage due to the target moving out of the radiation beam (Nill *et al* 2005). It is therefore physically impossible to generate a dose distribution with negligible dose outside the treatment target using photon radiation due to the presence of a low dose bath in the target’s surroundings.

We propose modifications to the margin concept, generating margins that are dependent on the beams’ incident directions. A new module was added to our in-house intensity-modulated radiation therapy (IMRT) treatment planning system DynaPlan (Kamerling *et al* 2016) to generate margins on a beam-by-beam basis, accounting for uncertainties perpendicular to beam incident directions. Our in-house IMRT treatment planning optimiser *μ*Konrad (Ziegenhein *et al* 2013) has been modified to use these beam-specific margins in the inverse planning process.

In this work, the PTV will no longer be defined in the traditional sense (i.e. in a binary manner where a voxel is either inside or outside the target volume) but is now dependent on the number and directions of the treatment beams. Probabilistic evaluation techniques are therefore employed for plan evaluation and comparison.

## 2. Methods

### 2.1. The modified margin concept

**Figure 1.**
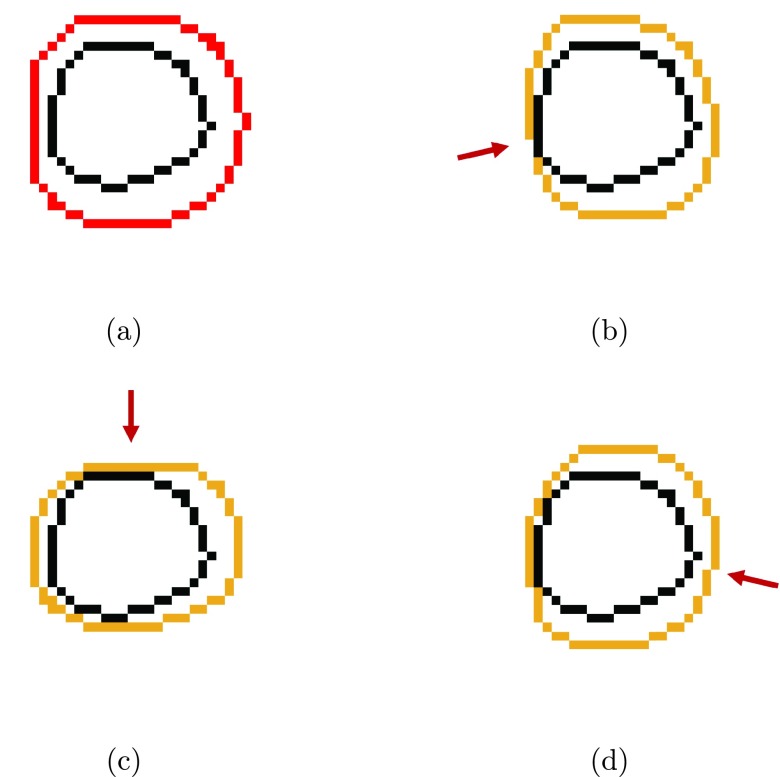
Isocenter axial contours of the prostate highlighted in black. (a) shows a PTV generated using van Herk’s margin recipe in red. (b)–(d) show beam direction dependent PTVs in orange, for gantry angles (b) 257°, (c) 0° and (d) 103°; beam directions are shown using red arrows.

The geometric uncertainties considered in this work are defined in the patient’s left-right, anterior-posterior and superior-inferior directions, and are assumed to be normally distributed with no correlations between them. The collapse of the trivariate Gaussian distribution, in beam direction, into a bivariate Gaussian distribution is accomplished analytically. To generate beam-dependent PTVs (bdPTVs), we expand each voxel within the CTV perpendicularly to the incident beam direction by an amount determined using the 2D version of van Herk’s margin recipe (VHMR), using the collapsed distribution as input parameters; the resulting bdPTV is the union of all voxel-based expansions. Figures 1(b)–(d) show the isocenter axial slices of the bdPTVs for one patient; figure 1(a) shows the isocenter axial slice of the PTV generated using VHMR for comparison.

We use an exclusive volume of interest (VOI) grid to define the structures used for treatment plan optimisation. This exclusive VOI grid defines only one VOI per voxel. In the event where VOIs overlap, for example the PTV for the prostate overlapping with the rectum, priority rules are used for voxel assignment. ICRU Report 83 (ICRU 2010) offers the option to subdivide the PTV into regions with different dose prescription to better spare nearby organs-at-risk (OARs); this technique will not be used in this work.

For use with arc therapy, the arc can be discretised into a finite number of control points, and treat each control point as a separate beam for margin-generation purposes. To perform inverse-planning with the new margin concept, modifications to the objective function used in the optimisation process are required.

### 2.2. Modifications to the objective function

The objective function minimised in *μ*Konrad is the standard piecewise quadratic objective function, shown in (2*a*)–(2*c*).

2*a* \begin{align*} E = \sum_i^{N_t} E_i^{\rm target} + \sum_j^{N_O} E_j^{\rm OAR} \label{eq:stdOF1} \nonumber \end{align*}

2*b* \begin{align*} E_i^{\rm target} = \sum_{k\in\mathcal{T}_i} \left\{s_i^u \left[d_i^{\rm min}-d_k\right]_+^2 + s_i^o \left[d_k-d_i^{\rm max} \right]_+^2 \right\} \label{eq:stdOF2} \nonumber \end{align*}

2*c* \begin{align*} E_j^{\rm OAR} = \sum_{k\in\mathcal{O}_j} s_j^o \left[d_k-d_j^{\rm max} \right]_+^2 \label{eq:stdOF3} \nonumber \end{align*}

The objective is calculated for each of the *N*_*t*_ targets and *N*_*O*_ OARs. Each VOI, subscript *i* for target and *j* for OARs, has its own user-defined penalty factors for underdosage $s_i^u$ and overdosage $s_i^o$, along with its respective dose thresholds $d_i^{\rm min}$ and $d_i^{\rm max}$. These penalties will only contribute to the objective if the dose *d*_*k*_ to voxel *k* within the VOI lies outside the permitted dose range. This is reflected by use of the positivity operator $[\bullet]_+$ and is defined as *[x]*_+_  =  *xH*(*x*), where *H*(*x*) is the heavyside step function.

For implementation purposes, the optimiser considers all penalty and threshold variables on a voxel-by-voxel basis. Each voxel therefore has its variables assigned prior to optimisation depending on which VOI the voxel belongs to within the exclusive VOI cube. The objectives that are used are shown in (3*a*)–(3*c*), where (3*b*) is the objective used for voxels belonging to any targets $\mathcal{T}$, and (3*c*) for all other VOIs, including OARs.

3*a* \begin{align*} E = \sum_{k\in\mathcal{T}} E_k^{\rm target} + \sum_{k\in\mathcal{O}} E_k^{\rm OAR} \label{eq:voxOF1} \nonumber \end{align*}

3*b* \begin{align*} E_k^{\rm target} = s_k^u \left[d_k^{\rm min}-d_k\right]_+^2 + s_k^o \left[d_k-d_k^{\rm max} \right]_+^2 \label{eq:voxOF2} \nonumber \end{align*}

3*c* \begin{align*} E_k^{\rm OAR} = s_k^o \left[d_k-d_k^{\rm max} \right]_+^2 \label{eq:voxOF3} \nonumber \end{align*}

**Figure 2.**
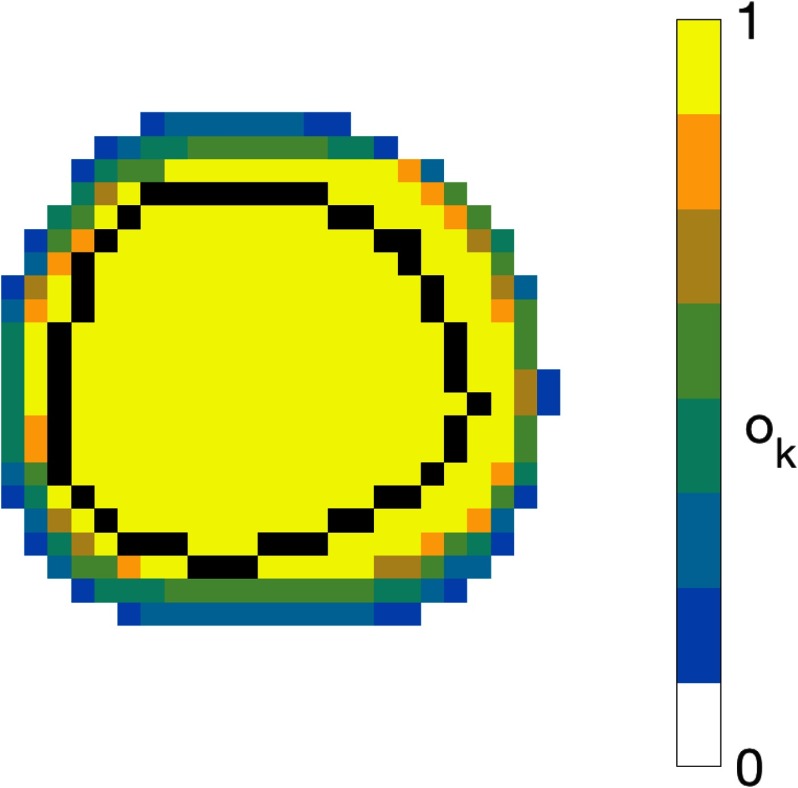
Isocenter axial slice showing the degree of overlap between the individual beam dependent PTVs for a treatment plan using 7 beam directions. The CTV is outlined in black.

The calculation of the objectives for beam dependent margins is done by repeating the objective calculation for the entire exclusive VOI cube for each beam direction. For speed considerations, the degree of overlap between the bdPTVs is used instead in the optimisation process. The overlap map is generated by summing and normalising the number of bdPTVs present in each voxel, and voxels where all beams overlap assume the value of 1. An example is shown in figure 2 demonstrating the variation between the overlap of the bdPTVs for a treatment plan using 7 beam directions.

The optimisation process now uses multiple VOI cubes. The first is the exclusive VOI cube describing the patient’s anatomy, without the use of any margins. The other VOI cubes describe the degree of overlap for each target, and provides a new variable $o_k^i$ for target *i* and voxel *k*. The modified objective function is shown in (4*a*)–(4*c*), and is evaluated for all voxels $\mathcal{E}$ within the external patient contour.

4*a* \begin{align*} E = \sum_{k\in\mathcal{E}} \left\{\sum_i^{N_t} o_k^i E_k^i + \left(1 - \sum_i^{N_t} o_k^i \right) E_k^{\rm OAR} \right\} \label{eq:overOF1} \nonumber \end{align*}

4*b* \begin{align*} E_k^i = s_i^u \left[d_i^{\rm min}-d_k\right]_+^2 + s_i^o \left[d_k-d_i^{\rm max} \right]_+^2 \label{eq:overOF2} \nonumber \end{align*}

4*c* \begin{align*} E_k^{\rm OAR} = s_k^o \left[d_k-d_k^{\rm max} \right]_+^2 \label{eq:overOF3} \nonumber \end{align*}

For voxels *k* with $o_k^i = 0, \forall i$, i.e. voxels with no beam dependent margins present, the first term of the objective function in (4*a*) reduces to 0, and we recover the original objective function (3*c*) for non-target voxels. On the other hand, voxels with $\sum\nolimits_i^{N_t} o_k^i &gt; 0$ implies contribution to one or more beam dependent margins/targets. Depending on how many beam dependent margins overlap in the specified voxel, i.e. how large $o_k^i$ is, the relative importance between the terms in (4*a*) will vary.

### 2.3. Evaluation tool and metrics

For the assessment of the margin size, we developed a probabilistic verification tool to model the effects of systematic and random geometric uncertainties using Monte Carlo techniques. Here, we assume the static dose cloud approximation to be reasonably accurate, where small displacements would not distort the dose distribution for tissue in regions of relatively homogeneous density (Craig *et al* 2003). The tool models the effects of the two types of uncertainty separately, and evaluates the probability that a certain DVH objective is achieved.

For each plan, we generate a population of identical CTVs. We shift the dose distribution for each CTV once by a displacement ${\bf a}_s$ following the trivariate Gaussian distribution describing the patient’s systematic uncertainty ${\bf \Sigma}$ in the left-right, anterior-posterior and superior-inferior directions. Then, for each sample, we shift the dose distribution by ${\bf b}_n$ following a distribution describing the patient’s random setup uncertainty ${\bf \sigma_r}$ for each fraction *n*. Trilinear interpolation is used to calculate the dose for all displacements. We then use the cumulative dose, across all *F* fractions, each sample receives for dose statistics calculations. The tool finally reports the fraction of the population which satisfies a given DVH objective.

Mathematically, the above can be expressed by (5), where $d({\bf x})$ describes the dose that is planned for delivery to a location at ${\bf x}$, and the cumulative dose we use for analysis denoted as $d_{c, s}({\bf x})$ for sample *s*.

5 \begin{align*} d_{c,s}({\bf x}) = \frac{1}{F} \sum_{n=1}^F d\left({\bf x}+{\bf a}_s+{\bf b}_n\right) \label{eq:evaluationTool} \nonumber \end{align*}

Using this tool, we can also generate dose volume coverage maps (DVCM) (Gordon *et al* 2010) by considering the DVHs generated for each sample to be solid under the curve, accumulating the scores for all samples, then dividing by the total number of samples used. The intensity of a point on this coverage map represents the probability that the DVH metric will be achieved on a population level.

In this paper, we will be using the conventional margins as the baseline for comparison to investigate how, on a population level, dose coverage for the various structures vary by using our novel per-beam margin concept.

### 2.4. Patient collection

In this planning study, five prostate patients were inverse-planned for IMRT treatments, with dose volume constraints following the PACE clinical trial recommendations (NCT01584258); all patients have given consent for their patient data to be used for research. Seven equidistance photon beams, at 6MV, were used, with the patients assuming a head-first supine position. The CTVs are prescribed 78Gy in 2Gy fractions. Target coverage was assumed to be satisfied if at least 98% of the volume received at least 95% of the prescribed dose.

The uncertainties used are assumed to follow Gaussians distributions, and the displacements are assumed to be uncorrelated between the left–right (LR), anterior–posterior (AP) and superior–inferior (SI) directions. The systematic uncertainties used are 1.1 mm, 1.1 mm and 1.5 mm in the LR, SI and AP directions, respectively, and 2.2 mm, 2.1 mm, 3.2 mm for the random uncertainties (McNair *et al* 2008). Voxel sizes of 1.91 mm, 1.91 mm and 2.5 mm in the LR, AP and SI directions are used. The CTVs are assumed to translate rigidly, and no rotations and deformations of the CTV are considered.

### 2.5. Planning comparison

We generate a set of plans for each patient: one using van Herk’s 3D margin recipe, and three plans using our per-beam margin concept with different max dose objectives to the rectum. Direct aperture optimisation is used to generate plans with 40 segments, using the implementation by Wild *et al* (2015). Optimisation objectives used are laid out in table 1. No attempts to refine and improve the plans are made in order to reduce the influence of the human element in radiotherapy treatment planning.

**Table 1.** IMRT inverse-planning optimisation objectives used to generate the treatments plans used in the planning study. The three levels of rectal DVH objectives are used to generate different plans using the beam-dependent margin concept.

Organ	Function	Dose/Gy	Weight
Prostate (PTV)	Min dose	74.5	10
	Max dose	82.0	10
Rectum	Max dose	70.0/65.0/60.0	8
Bladder	Max dose	70.0	6
Femoral heads	Max dose	50.0	2
External	Max dose	60.0	2

Apart from the changes to the rectal max dose objective, all other planning objectives are fixed. This is to better understand how the optimiser responds to changes in planning objectives, as it would be extremely difficult to find tradeoffs when modifying multiple variables at the same time.

The evaluation tool is applied to all plans, using a population size of 50 000. A selection of DVH criteria were chosen for planning comparison: 98% of the target volume ($D_{98\%}$) to receive at least 95% of the prescribed dose, as representative of dose coverage; the volume of rectum receiving 70 Gy and 75 Gy should not exceed 5% and 2% respectively, to be representative of high dose to the rectum; and the volume of bladder receiving 74 Gy should not exceed 2%, to be representative of high dose to the bladder. DVCMs are generated for the rectum, again using a population size of 50 000. For comparison purposes, dose volume coverage difference maps (DVCDMs) are generated by subtracting DVCMs for plans using beam dependent margins from the DVCM for the treatment plan generated using conventional margins.

## 3. Results

Table 2 lists the volume of the conventional PTVs and the union of bdPTVs for all patients. As the union of bdPTVs include voxels with $o_k^i &lt; 1$, the volume of the union of bdPTVs over-represents the region of high does that is required to be delivered to satisfy planning requirements.

**Table 2.** Volumes for conventional PTVs and the union of bdPTVs for all patients.

Patient	Volume/cm^3^		
	3D PTV	2D bdPTVs	Difference (%)
1	99.27	93.97	−5.34
2	99.66	96.99	−1.68
3	81.57	75.50	−7.43
4	108.02	102.81	−4.82
5	90.24	85.71	−5.02

**Figure 3.**
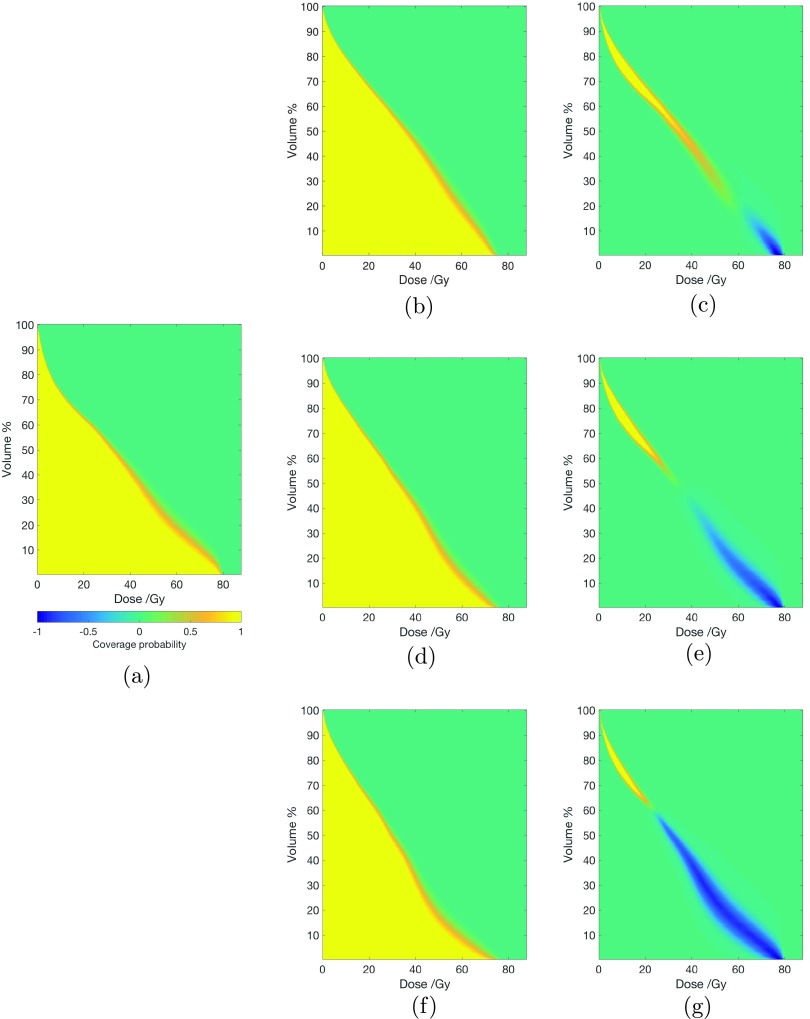
(a) Dose volume coverage map (DVCM) for the rectum, for a treatment plan using conventional margins, generated using a population size of 50000. The colour scale is used for all figures, and represents the probability that the DVH metric is delivered to the patient. Negative values are only used in dose volume coverage difference maps (DVCDMs). ((b), (d) and (f)): DVCMs for the rectum for treatment plans using our beam dependent margin concept, planned using max rectal dose objectives at 70 Gy, 65 Gy and 60 Gy, respectively. A population size of 50 000 was used for generating the results. ((c), (e) and (g)): DVCDMs showing the difference in dose volume coverage between the treatment plan using conventional margins and the various treatment plans using our beam dependent margin concept at different max rectal dose objectives.

Results from the probabilistic verification tool can be found in tables 3–5, for the prostate (CTV), rectum and bladder respectively. The values show the probability that the specified dose volume objective is satisfied. Figure 3 shows the dose volume coverage maps for the rectum. Here, the values represent the probability that the dose volume point is reached. DVCDMs showing the difference between the treatment plan using conventional margins and treatment plans using our beam dependent margin concept are also included.

**Table 3.** Results from the verification tool, using a population size of 50 000. The values show the probability in percentages of the CTV receiving at least 95% of the prescribed dose to 98% of the volume, to two decimal places. The suffixes R70, R65 and R60 represent plans using 70 Gy, 65 Gy and 60 Gy as the max dose objective for the rectum, respectively.

Patient	CTV: $D_{98\%}$ > 95% *D*_pres_			
	3D_R70	2D_R70	2D_R65	2D_R60
1	99.99	99.11	96.69	95.39
2	99.99	99.91	99.90	99.84
3	100.00	98.20	93.67	89.90
4	100.00	99.90	99.61	99.01
5	99.99	98.90	99.72	99.34

**Table 4.** Results from the verification tool, using a population size of 50 000. The values show the probability in percentages of the rectum satisfying the DVH objectives, to two decimal places. The suffixes R70, R65 and R60 represent the max dose objective for the rectum at 70 Gy, 65 Gy and 60 Gy, respectively.

Patient	Rectum: $V_{70~{\rm Gy}}$ < 5%				Rectum: $V_{75~{\rm Gy}}$ < 2%			
	3D_R70	2D_R70	2D_R65	2D_R60	3D_R70	2D_R70	2D_R65	2D_R60
1	0.64	11.76	60.82	82.85	0.42	81.78	87.52	88.36
2	3.83	11.55	47.29	69.07	1.94	30.01	46.84	49.28
3	1.24	20.81	65.41	84.61	1.44	90.95	92.31	94.44
4	0.02	1.34	13.15	31.80	0.08	14.38	25.33	39.00
5	3.64	31.96	76.21	89.67	6.48	83.05	90.63	93.38

**Table 5.** Results from the verification tool, using a population size of 50 000. The values show the probability in percentages of the bladder receiving no more than 74 Gy to 2% of the volume, to two decimal places. The suffixes R70, R65 and R60 represent the max dose objective for the rectum at 70 Gy, 65 Gy and 60 Gy, respectively.

Patient	Bladder, $V_{74~{\rm Gy}}$ < 2%			
	3D_R70	2D_R70	2D_R65	2D_R60
1	13.00	55.95	51.56	51.34
2	2.06	10.07	9.41	11.69
3	70.99	94.40	93.76	94.18
4	51.42	90.85	89.93	89.85
5	9.20	14.04	12.04	10.76

## 4. Discussion

Both van Herk’s margin recipe and our beam dependent margin concept share the assumptions: the uncertainties follow a Gaussian distribution, and only rigid translations of the treatment target requires consideration. The effects of rotations and deformations of the target are not accounted for in this study.

This planning study aims to address the inability to physically produce perfectly conforming dose distributions using photon radiation, as there will also be a presence of entrance and exit doses due to how photons interact with matter. We tackle this by considering only uncertainties on a beam-by-beam basis, accounting for uncertainties perpendicular to beam incident directions. This takes the assumption that small movements in beam direction result in negligible difference in dose, considering the exponential relationship between absorbed dose and radiological depth.

We assume dose invariance to hold for all generated scenarios of geometrics shifts of the anatomy in this planning study. For the prostate, this assumption holds reasonably well, and has been used by other authors as well (Poulsen *et al* 2012, Fast *et al* 2014, 2016, Colvill *et al* 2015). For sites where electron density inhomogeneity is dominant or has significant amount of air/tissue interfaces, full Monte Carlo dose calculations would be required for accuracy (Witte *et al* 2014).

The per-beam margin concept is able to reduce the amount of high dose delivered to the rectum, at the cost of delivering additional low dose as a tradeoff. We are able to maintain sufficient target coverage, albeit slightly lower when compared to conventional margins, as can be seen in table 3. This implies that conventional margins are more conservative than required.

We also observe similar behaviour for dose to the bladder. Moreover, as we aim to decrease the maximum dose to the rectum, it results in increases in dose to the bladder. This is a direct consequence of the optimiser’s aim to maintain sufficient dose to the target whilst reducing dose the rectum, resulting in its redistributing dose to other OARs. This tradeoff occurred as no changes to the bladder’s optimisation objectives were made.

Another feature of the modified margin concept is the increased control over the finite dose gradient in the peripheral region of the target. The use of conventional margins effectively requests the optimiser to produce the steepest dose gradient possible at the edge of the PTV. Our beam-dependent margin concept, on the other hand, requests a more achievable dose gradient in the form of the overlap map, such as the example shown in figure 2. For regions where the PTV overlaps surrounding OARs, this additional information allows the optimiser to better find compromises between the conflicting objectives. This is in contrast to the use of ring structures, where the user can make use of several dose levels alongside derived regions of interest (ROIs) to control the different severity of dose fall-off in the regions surrounding the PTV. The overlap map simplifies this by using the relative optimisation weights for the structures as parameters on the degree of compromise between the VOIs when generating the dose fall-offs.

An obstacle to probabilistic planning is the lack of definitive guidelines during the planning process. Current clinical practice employs the use of various metrics, such as $D_{2\%}$ (or *D*_min_), $D_{98\%}$ (or *D*_max_) and median dose ($D_{50\%}$) to the target, as surrogates to indicate whether sufficient target coverage and dose homogeneity are achieved. For OARs, various DVH points are used to estimate the probability of complications and toxicity that the patient may encounter. However, in probabilistic planning, coverage probability is usually determined by simulating the effects of the uncertainty in the plan evaluation process, as demonstrated by the verification tool employed in this study. Whilst it is possible to use the results from a probabilistic evaluation tool to feedback into the planning optimisation step, the duration of the optimisation should be taken into consideration for clinical implementation.

Exploiting the optimiser’s dependency on the degree of overlap, the ability to extend the margin concept to consider relative placement of nearby organs at risks should be investigated. In order to shape the PTV away from the OAR, the PTV would generally need to be larger in order to maintain sufficient target coverage. The small degree of overlap, i.e. a low $o_k^i$ value, in the peripheral regions of the margin will contribute less towards the objective. This should allow for more personalised treatment plans to further reduce the probability of side effects occurring.

## 5. Conclusion

A probabilistic verification tool was created to simulate the effects of systematic and random uncertainties. Using this tool, we demonstrated how the use of beam dependent margins can decrease the amount of high dose delivered to OARs that are in close proximity to the CTV, whilst maintaining a high level of target coverage. There exists a tradeoff between delivering less high dose and additional low doses to the OARs by using beam dependent margins; this is a consequence of the optimiser solving the modified optimisation problem and redistributing the total dose delivered to the patient.
